# One Step Back from Bedside to the Bench—How Do Different Arterial Stiffness Parameters Behave in Relation to Peripheral Resistance?

**DOI:** 10.3390/diagnostics13182897

**Published:** 2023-09-09

**Authors:** Nóra Obajed Al-Ali, Sára Rebeka Tóth, László Váróczy, László Imre Pinczés, Pál Soltész, Zoltán Szekanecz, György Kerekes

**Affiliations:** 1 Division of Hematology, Department of Internal Medicine, Faculty of Medicine, University of Debrecen, 4032 Debrecen, Hungary; laszlo.varoczy@gmail.com (L.V.); pinczes.laszlo.imre@med.unideb.hu (L.I.P.); 2 Department of Cardiology, Medical Centre, Hungarian Defence Forces, 1134 Budapest, Hungary; sasare1991@gmail.com; 3 Division of Angiology, Department of Internal Medicine, Faculty of Medicine, University of Debrecen, 4032 Debrecen, Hungary; dr.soltesz.pal@gmail.com; 4 Department of Rheumatology, Faculty of Medicine, University of Debrecen, 4032 Debrecen, Hungary; szekanecz@gmail.com; 5 Division of Intensive Care, Department of Internal Medicine, Faculty of Medicine, University of Debrecen, 4032 Debrecen, Hungary; gkerekes@med.unideb.hu

**Keywords:** arterial stiffness, cardiovascular risk, elasticity parameters, distensibility coefficient, pulse wave velocity, oscillometry, applanation tonometry

## Abstract

The investigation of arterial stiffening is a promising approach to estimating cardiovascular risk. Despite the widespread use of different methods, the dynamic nature of measured and calculated stiffness parameters is marginally investigated. We aimed to determine the stability of large artery elasticity parameters assessed via commonly used, ultrasound-based and oscillometric methods in relation to peripheral resistance modulation. A human experimental environment was composed, and fifteen young males were investigated at rest after extremity heating and external compression. Functional vascular parameters were monitored in each session, and several arterial stiffness parameters were analysed. The distensibility coefficient (DC) did not show significant changes during heat provocation and extremity compression, while DC’s stability seemed to be acceptable. The same stability of carotid–femoral pulse wave velocity (PWV) was detected with ultrasound measurement (5.43 ± 0.79, 5.32 ± 0.86 and 5.28 ± 0.77, with *p* = 0.38, *p* = 0.27 and *p* = 0.76, respectively) with excellent intersession variability (intraclass correlation coefficient of 0.90, 0.88 and 0.91, respectively). However, the oscillometric PWV (oPWV) did change significantly between the heating and outer compression phase of the study (7.46 ± 1.37, 7.10 ± 1.18 and 7.60 ± 1.21, with *p* = 0.05, *p* = 0.68 and *p* < 0.001, respectively), the alteration of which is closely related to wave reflection, represented by the changes in reflection time. Our results indicate the good stability of directly measured elastic parameters such as DC and PWV, despite the extreme modulation of peripheral resistance. However, the oscillometric, indirectly detected PWV might be altered by physical interventions, which depend on wave reflection. The effective modulation of wave reflection was characterized by changes in the augmentation index, detected using both oscillometry and applanation tonometry. Thus, the environment during oscillometric measurement should be rigorously standardized. Furthermore, our results suggest the dynamic nature of the reflection point, rather than being a fixed anatomical point, proposed previously as aortic bifurcation.

## 1. Introduction

Arterial stiffness parameters as clinically accessible measures of vascular ageing are promising tools for cardiovascular (CV) risk estimation [1,2]. Despite the earlier optimism leading to the early inclusion of pulse wave velocity (PWV) into CV risk estimation [3], no clinical guidelines were developed at the beginning of the 2020s that firmly recommended these methods as a necessary part of total or additional CV risk stratification [4,5,6]. Numerous innovative methods are available on the market or in experimental settings, leading to significant inconsistency [7]. Therefore, despite large population-based prospective studies, the stiffness parameters are withheld from current recommendations, mainly due to the lack of evidence in reclassification potential [8,9]. On the other hand, 30 years without widespread breakthroughs suggests the need for the renewal of basic research; namely, the isolated elastic properties of central vessels may be adequately measured without the influence of peripheral arterial resistance [10].

Among others, two main approaches are mainly implemented to estimate the central PWV. During direct “two-point” measurement methods, the pulse wave propagation is detected over different points of the body either with direct contact (applanation tonometry) or via pulse-wave-dependent parameters (Pulsatile Doppler or magnetic resonance). The place of accessible measurement points is the main weakness of this methodology, leading to the inaccuracy of the estimation of the travelled path. On the other hand, the indirect “one-point” measurement is an attractive method for using the brachial artery as a natural aortic arch cannula to detect the delay of the reflected wave in relation to the primary aortic wave, assuming aortic bifurcation as the reflection point. Thus, the “one-point” measurement provides a handy methodology with doubled pitfalls; the superficial estimation of travelled distance and doubtfulness in the reflection point may potentially modify the results [11].

All the hemodynamic models comprise arterial resistance (resistor) and arterial compliance (capacitor) in parallel relation, assuming that changes in arterial resistance do alter the compliance of large elastic vessels [12]. According to this model, the in vivo measured human parameters representing central arterial elasticity may be influenced by peripheral resistance, leading to the significant instability of former parameters in real-life conditions. Therefore, we hypothesized that extremes of physiological peripheral resistance changes could alter central arterial capacitance and, thus, elastic properties characterized by some aortic stiffness parameters. Furthermore, we aimed to determine the stability of the presumed “static” aortic reflection point during “one-point” measurement in connection with physical interventions affecting peripheral resistance. Thus, the first objective has been to detect any significant changes in different stiffness parameters in different resistive conditions. The second objective, in the case of non-significant changes, was to determine the agreement of different stiffness parameters after the modulation of peripheral resistance.

## 2. Materials and Methods

### 2.1. Study Population and Environmental Settings

First, a human experimental milieu was created to improve study sensitivity. According to this goal, a relatively homogeneous human study population was recruited. Fifteen young (mean age 22, 21–25 years), healthy, non-obese (body mass index 23.1 ± 1.02 kg/m^2^), average body build (body height 180.3 ± 4.8 cm), non-smoker, normotensive (manually measured systolic blood pressure 118.7 ± 7.2, diastolic blood pressure 77.3 ± 4.6 millimetres of mercury—mmHg), male medical student subjects were involved to avoid hormonal and environmental influences. Alcohol, caffeine, vasoactive drug, and painkiller intake was prohibited 24 h before the vascular assessment. The study was performed between 8 and 10 h in the morning after 12 h of starvation in a quiet, dark, air-conditioned room at 21 °C, after 30 min of accommodation in a supine lying position. 

### 2.2. Vascular Assessments

All the superficial measurement points were marked, and repeated measurements were carried out at the same point to avoid the influence of distance determination on final calculations. The raw data of all stiffness detection methods were digitally recorded and stored, and their analysis and final calculations were performed offline.

#### 2.2.1. Oscillometric Method

The “one-point” arterial stiffness measurements were carried out using the oscillometric method (Arteriograph, TensioMed LTD, Budapest, Hungary) over the right brachial artery of every subject. The primary measurement methodology was described elsewhere [13]. In brief, firstly, the distance between the suprasternal notch and the symphysis was measured to calculate the travelled distance of the pulse wave. Subsequently, a sphygmomanometric cuff was placed on the right upper arm, and suprasystolic pressure was maintained to obtain oscillometric pressure signals. This device determines the reflection time (RT) between the primary systolic peak and the first reflected wave, representing the aggregate of the pulse’s travel time forward and backward in the aorta below the origin of the brachial artery. Finally, the device calculates the oscillometric PWV (oPWV) as a ratio of travelled distance and half of the reflection time. Furthermore, other wave-reflection-based and central blood pressure parameters were automatically detected and calculated at rest and after every physical intervention affecting peripheral resistance, such as the augmentation index (oAix) and central blood pressure (oCBP).

#### 2.2.2. Radial Artery Applanation Tonometry

A Sphygmocor device (SphygmoCor 8.0, AtCor Medical, Sydney, Australia) was introduced to obtain the central pressure waveform. Although the device can record both carotid and femoral waveforms, to avoid errors due to sequential placement, validated radial arterial tonometry with generalized transfer function was continuously recorded at the same point over the right radial artery to detect the central arterial waveform [14,15,16]. All the measured and calculated stiffness parameters resulted from 10 averaged heart cycles. Among others, ejection duration (ED), the time to the peak of the first (T1) and second pressure wave components (T2), the time to return (Tr), aortic pulse pressure (aPP), augmentation pressure (AP), augmentation index (aAix), and heart-rate-corrected aAix adjusted to a heart rate were displayed and used for statistical analysis. In addition, the central PP results at the time of carotid M-mode ultrasound recording were utilized as ∆p for calculating carotid distensibility.

#### 2.2.3. Ultrasound-Based Methods

The “two-point” PWV was determined as carotid–femoral PWV (cfPWV), using ultrasound-based electrocardiography (ECG)-gated pulsed-Doppler (PD) analysis over the left common carotid and right femoral arteries; the details of the methodology were described previously [17]. After determining and marking carotid and femoral measurement points, the distances between the suprasternal notch and the two sampling sites were measured, and the travelled path of the pulse wave was calculated. Subsequently, six cycles of ECG-gated Doppler signals were recorded over each sampling site, and the time delay between carotid and femoral Doppler signals was calculated using the foot-to-foot method. The cfPWV was defined as a ratio of travelled distance and time delay. The cfPWV was measured and calculated using ultrasound equipment (Philips HD 11 XE, L12-3 linear array transducer). The same ultrasound equipment was applied for direct distensibility calculations of the common carotid artery [11]. A cross-sectional, magnified view was obtained from the left common carotid artery at the same point as the previous PD Doppler analysis. The ultrasound beam was carefully tilted until clear anterior and posterior double lumen-intima and media-adventitia borders were acquired; then, midline perpendicular ECG-gated M-mode pictures were recorded and digitally stored for further distensibility calculations. Six carotid M-mode cycles were used for common carotid artery diameter measurements. The average of different diameters was used for carotid distensibility (DC) determined by the equation DC = (2∆d/d)/(∆p), where DC means the coefficient of distensibility, d is the diastolic diameter, Δd is the difference between systolic and diastolic diameters, and Δp is the central pulse pressure determined using the Sphygmocor method [18]. 

### 2.3. Study Design 

**Figure 1:**
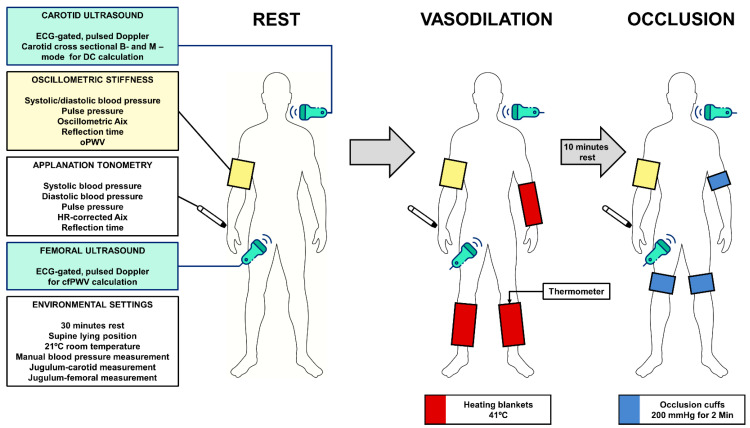
Flowchart of the methods for the investigation of arterial stiffness parameters. ECG—electrocardiogram, DC—distensibility coefficient, Aix—augmentation index, oPWV—oscillometric pulse wave velocity, HR—heart rate, cfPWV—carotid–femoral pulse wave velocity, °C—degrees of Celsius, mmHg—millimetres of mercury, Min—minutes.

Every subject was comfortably placed in a supine position, and an oscillometric cuff was placed on the right upper extremity. Then, the right wrist was fixed for radial applanation tonometry (SphygmoCor). ECG electrodes were attached, and commercially available heating blankets (HK5510, AEG, Nürnberg, Germany) were placed bilaterally around both the crural and left forearm regions. A thermometer was placed on the skin at the upper border of the blanket (Periflux PF 5020, Perimed AD, Stockholm, Sweden). The carotid and femoral measurement points were defined and marked, and all the travelled distances were measured with a calliper. After 30 min of rest, the right arm’s brachial blood pressure was determined manually to calibrate radial tonometry, and then, the blood pressure cuff was replaced with the oscillometric stiffness device (Arteriograph). At rest and later, after different physical interventions, the following investigations were carried out in a predetermined sequence by two well-trained investigators (S.R.T. and Gy.K.): the simultaneous acquisition of M-mode common carotid recordings and radial artery tonometry, then pulse wave Doppler recordings of the common carotid and common femoral arteries and repeated oscillometric pulse wave analysis and PWV measurements. To modulate the peripheral resistance, two opposing physical interventions were carried out as provocation manoeuvres. To induce lower-extremity vasodilation, both heating blankets were activated, and after reaching skin temperature of 41 °C, all the vascular assessments were repeated using the former sequence. Subsequently, the heating blankets were removed, and after 10 min of rest, large pneumatic tourniquet cuffs were placed over both knees and above the elbow on the left side, and suprasystolic 200 mmHg was generated and fixed for the complete occlusion of the femoral and left brachial arteries. The previously specified vascular assessments were performed to reach the hemodynamic redistribution after 2 min of occlusion (Figure 1).

### 2.4. Statistical Analysis

Categorical variables are given as their frequencies and percentages, while continuous variables are given with means and standard deviations (SDs). The Shapiro–Wilk test was used for the evaluation of data normality. Student’s paired *t*-test or the Wilcoxon signed rank test was applied to compare the same stiffness parameters at rest and after every provocation manoeuvre based on data normality. In the case of non-significant changes, agreement investigations—intraclass correlation coefficient (ICC) and the Bland–Altman difference plotting method—were performed to prove the stability of different stiffness parameters. We also employed a simple linear regression analysis to examine the relationship between PWV measurement methods during different provocation manoeuvres. The level of statistical significance was considered at *p* < 0.05. Statistical analyses were performed using SPSS 26.0 (IBM Corp., Armonk, NY, USA).

## 3. Results

All the ultrasound, oscillometric and applanation-based methods were compared at rest, during heat provocation and during external compression. Then, both ICC and Bland–Altman plots were determined between every session to reveal the stability of different stiffness parameters and unmask the statistical pitfalls of the former analysis. The results are summarized in Table 1 and Supplementary Tables S1–S3.

### 3.1. Direct Ultrasound Measurement

**Figure 2:**
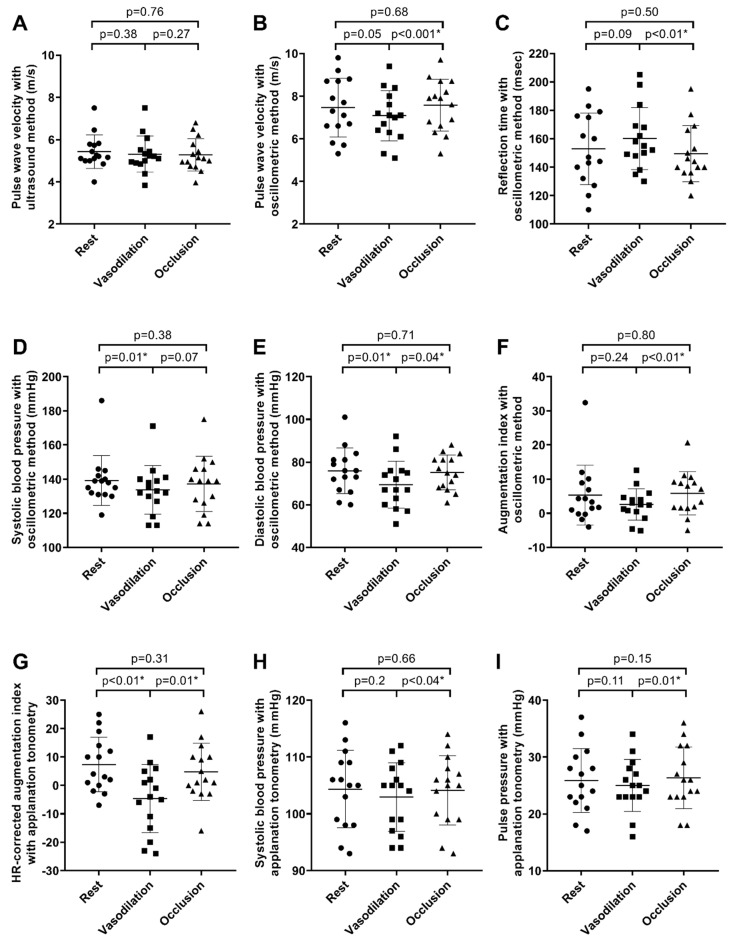
The effects of vasodilation and occlusion on arterial stiffness parameters (**A**–**I**). Each dot represents an individual participant’s data. The horizontal line in the centre of the plot represents the mean score for the group, while the vertical lines extending from the horizontal line represent one standard deviation above and below the mean. The * signs indicate significant results of Student’s paired *t*-test or the Wilcoxon signed rank test, based on data normality. m/s—metre per second, msec—millisecond, mmHg—millimetres of mercury, HR—heart rate.

**Figure 3:**
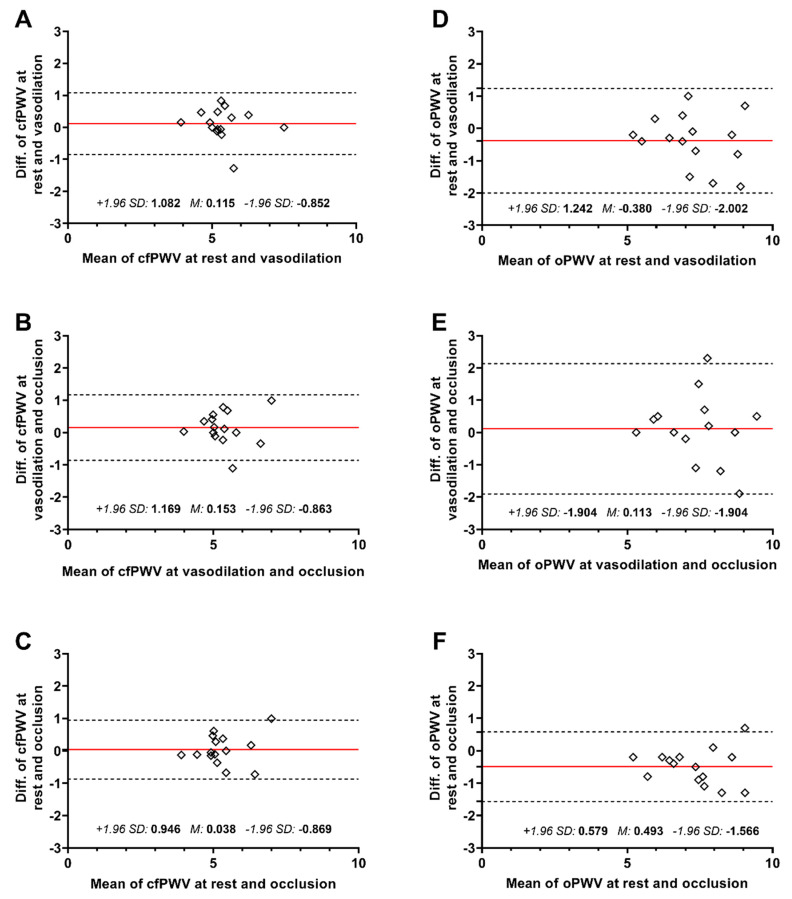
Bland–Altman plots. The solid redline represents the mean bias, whereas the dashed lines indicate the limits of agreement computed as the mean bias plus or minus 1.96 times its standard deviation. Data in (**A**–**C**) are from direct ultrasound measurements, whereas data in (**D**–**F**) are from the indirect oscillometric method. Diff.—difference, cfPWV—carotid–femoral pulse wave velocity, oPWV—oscillometric pulse wave velocity, SD—standard deviation, M—mean bias.

**Figure 4:**
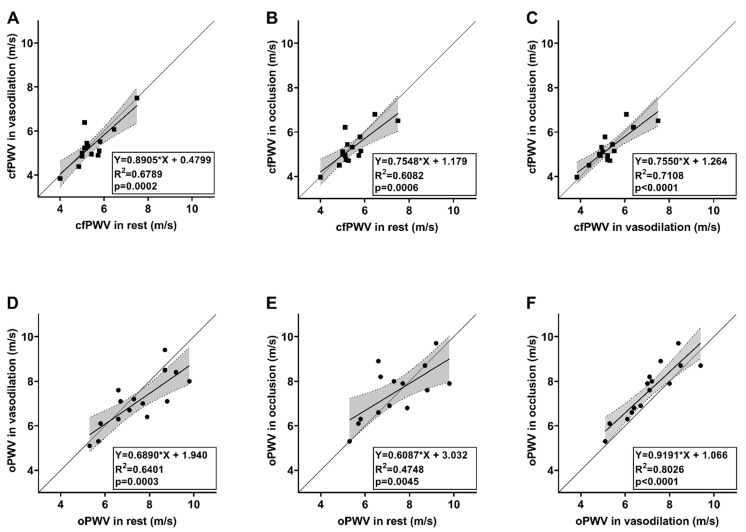
Scatter plots illustrating the relationship between direct ultrasound measurements and oscillometric measurements with simple linear regression analysis (**A**–**F**). The scatter plot displays the data points representing the variables. The regression line (bold solid line) represents the best-fit linear relationship between the variables, while the non-bold line equals the line of identity. Dotted lines represent confidence intervals for the regression line. The equation of the regression line is y = mx + b, where m is the slope and b is the y-intercept. cfPWV—carotid-femoral pulse wave velocity, oPWV—oscillometric pulse wave velocity, m/s—metre per second, R^2^—coefficient of determination.

The carotid distensibility coefficient, via direct ultrasound measurement and applanation tonometry, did not change significantly over the three measurements, and the ICC and Bland–Altman plots confirmed good reliability without any trends. Also, carotid–femoral pulse wave velocity changes were not significant over the three measurements (5.43 ± 0.79, 5.32 ± 0.86 and 5.28 ± 0.77, with *p* = 0.38, *p* = 0.27 and *p* = 0.76, respectively), with excellent reliability, and without the tendency of over- or underestimation (Figure 2A and Figure 3A–C). The slopes of cfPWV regression lines between each session were above 0.75, representing the excellent stability of this method (Figure 4A–C). Therefore, it can be concluded that changes in peripheral resistance do not significantly influence the direct measurement of ultrasound-based arterial stiffness parameters.

### 3.2. Indirect Oscillometric Method (Arteriograph)

PWV, determined indirectly via the oscillometric method, decreased with borderline significance during heat provocation (7.46 ± 1.37 and 7.10 ± 1.18, *p* = 0.05) and re-increased significantly during compression, compared to the vasodilated state (7.10 ± 1.18 and 7.60 ± 1.21, *p* < 0.001) (Figure 2B). Despite the excellent reliability determined by ICC, the Bland–Altman plot revealed a not negligible underestimation of PWV during heat provocation (Figure 3D–F). The linear regression analysis confirmed an altered slope of oPWV lines depending on vasodilation and occlusion (Figure 4D–F). In line with former changes, reflection times increased during vasodilation (152.90 ± 25.25 and 160.10 ± 21.80, *p* = 0.09) and then decreased significantly with the re-increase in peripheral resistance (160.10 ± 21.80 and 149.50 ± 19.68, *p* ≤ 0.01) (Figure 2C). Systolic and diastolic blood pressures decreased during vasodilation (139.20 ± 14.62 and 133.80 ± 14.21, *p* = 0.01; 75.90 ± 10.65 and 69.40 ± 11.02, *p* = 0.01, respectively), while diastolic blood pressure elevated during external mechanical compression (69.40 ± 11.02 and 75.10 ± 8.12, *p* = 0.04) (Figure 2D,E). The ICC results indicated excellent systolic, diastolic and pulse pressure reliability. The augmentation index elevated after the re-increase in peripheral vascular resistance (2.60 ± 4.57 and 5.80 ± 6.32, *p* < 0.01), while the stability of this index seemed to be insufficient during heat provocation (5.30 ± 8.74 and 2.60 ± 4.57, *p* = 0.24) (Figure 2F). No significant changes in ED, PP and aPP were observed with the indirect oscillometric method. 

In order to elucidate the relation between the changes in oPWV and the changes in the potentially wave-reflection-dependent parameters, a correlation analysis was carried out. Changes in oPWV were strongly and significantly correlated with changes in RT between every study session. However, there was a weak correlation between oPWV and the oscillometric augmentation index (oAix), indicating the complexity of this parameter (Supplementary Table S4).

### 3.3. Applanation Tonometry (Sphygmocor)

Heart-rate-corrected augmentation index values determined via applanation tonometry changed significantly both during vasodilation (7.30 ± 9.62 and −4.70 ± 12.02, *p* ≤ 0.001) and external mechanical compression (−4.70 ± 12.02 and 4.70 ± 10.05, *p* = 0.007). During the third measurement, values returned to baseline (Figure 2G). The ICC results indicated moderate applanation-based augmentation index reliability during peripheral resistance manipulation. Systolic blood pressure and pulse pressure increased significantly during external mechanical compression compared to heat provocation (102.90 ± 6.02 and 104.10 ± 6.10, *p* = 0.04; 25.00 ± 4.58 and 26.30 ± 5.39, *p* = 0.01, respectively) (Figure 2H,I). The applanation tonometry showed the best reliability regarding central blood pressure parameters. No relevant changes in diastolic blood pressure, reflection time and ejection duration were observed. The reliability of reflection time was unacceptable.

## 4. Discussion

Some profound physical interventions, such as heating the skin or the external compression of peripheral arteries, might result in a significant decrease or increase in peripheral vascular resistance, leading to a significant decrease or increase in Aix, respectively. These vascular changes may be detected via oscillometric methods and applanation tonometry. The prolongation and shortening of reflection time is the primary driver of central pulse wave morphological changes, which are believed to be mainly dependent on pulse wave velocity. Earlier pharmacological interventions such as inhaled β-mimetics or intravenous nitrates provoked the same drop in arterial resistance and a subsequent Aix decrease in previous interventional studies [19,20]. Therefore, our results support the previous suggestions that a decrease in peripheral resistance may alter wave reflection and central blood pressure augmentation.

In contrast to pharmacological vasodilation, the thermic effect of the environment is a continuous variable. Therefore, only the standardization of room temperature may prevent interference. Not surprisingly, as the arterial circulation of extremities is a high resistance system at rest, the external compression of upper and lower extremity arteries did not significantly increase Aix compared to baseline; it just potentiated the high baseline resistance. However, more sophisticated experimental methods revealed earlier wave reflection even if the external compression was lower in intensity or a single extremity was affected [21,22].

Both theoretical models derived from resistors and capacitors in parallel relation [12] and hemodynamic animal models [23] indicate a significant effect of peripheral resistance or blood pressure on central arterial elasticity, respectively. This experimental finding has been supported by population-based trials [24,25], but the chronic morphologic and functional changes due to hypertension and acute effects due to wall tension cannot be separated easily [26]. In this young, healthy male population, systolic and diastolic central blood pressures decreased significantly, but not pulse pressures, detected via the oscillometric method after peripheral vasodilation. On the contrary, the “gold-standard” applanation tonometry detected mild but significant systolic and pulse pressure re-increases between the vasodilation and occlusion stage. Despite the extreme heat load causing a decrease in systolic and pulse pressure, no significant change was observed, and there was excellent agreement between the capacitor represented by the distensibility coefficient and ultrasound-based cfPWV. This result underlies the earlier physiological finding that peripheral resistance is not a significant determinant of peripheral impedance and targets organ damage in chronic vascular pathology [27]. Furthermore, our results indicate a minor role of pulse wave velocity and, thus, wall elasticity in dynamic changes in the central augmentation index.

In contrast to ultrasound-based methods, oPWV has shown a reflection-time-dependent, nearly significant decrease during heat provocation and a significant re-increase after extremity compression. The agreement analysis revealed a consequent underestimation of PWV during heat provocation without notable result dispersion. The one-point PWV measurement precludes a fixed anatomical aortic reflection point for PWV calculation. However, the “gold-standard” two-point-dependent cfPWV did not change during provocations, suggesting the constant nature of large arterial elasticity in this homogenous population during the physical modulation of resistance. In contrast, in invasive rabbit models, Horikoshi et al. proved a decrease in stiffness in muscular arteries and an opposite increase in aortic stiffness during continuous systemic, intravenous dihydropyridine (DHP)-type calcium channel blocking agent or phentolamine administration [28,29]. Nevertheless, the systemic DHP-type calcium channel blocking agent treatment in humans resulted in decreased integrated elastic and muscular stiffness, represented by the cardio-ankle vascular index [30]. The main difference between the abovementioned and our studies is the systemic effect of drug administration, compared to the local effect of heat provocation and arterial occlusion. Also, the difference in the proportion of muscular and elastic arteries in rabbits and humans is due to the different extremity/torso ratios. This inconsistency in peripheral–central stiffness inter-relation further increases the need to develop an invasive model on human or primate subjects. 

Accepting our previously proven stable nature of cfPWV and carotid distensibility, the decrease in oPWV during heat provocation must depend on the wandering of the reflection point. Counting back from means of oPWV changes on average, around 3.75 cm displacement of the reflection point may be calculated between the two provocation manoeuvres, which composes a 7.5% change of mean measured distance. This result indicates that oPWV is a complex, functional vascular parameter rather than a single indicator of aortic elasticity and that oPWV is significantly influenced by wave reflection. 

As a result of our findings, at least a warning should be considered to firmly uphold the standardization of the environment during a single oscillometric functional vascular assessment. Furthermore, although there is no available information about the influence of 24 h long recording and oscillometric data analysis on PWV instability, the statistical approach of large amounts of variables may eliminate the confounding factors.

As a primary conclusion, our results suggest that different methods are not interchangeable despite the same detected variable. Furthermore, the indirect “one-point” measurements are generally more unstable due to the wave reflection dependency. Thus, both method-specific normal values and rigorous standardization of the environment should be introduced. 

In contrast to the basic concept of reflection-based stiffness measurement methods, the reflection point seems more of a theoretical functional point with a dynamic nature than an actual anatomic point. However, our investigation proved that this flexibility of wave reflection is not negligible, which must be considered both in the standardization of the environment and in advance of stiffness innovation.

Deeply absorbed in the physiology of arterial stiffness, the dynamic nature of arterial stiffness parameters, e.g., wandering of theoretical wave reflection point, and the inter-relation of resistivity and capacitance and wave reflection are widely but not sufficiently explored in basic research [31]. Nevertheless, valid information from this field may prevent the delay of some promising methods to be involved in individual atherosclerotic risk stratification. Furthermore, despite the continuous transition of elastic arteries into muscular and more resistive arteries, the level of arterial stiffness measurements and the accurate definition of arterial stiffness must be determined. Some authors adhere to the great elastic arteries, while others are widening the stiffness idea [7,11,32,33]. Thus, confounding factors originating from radical innovation in vascular methodologies—such as the involvement of peripheral vascular beds—may be prevented promptly.

Our study may indeed have some limitations. Aiming to collect significant results from a relatively small sample size, the involved patient population was relatively homogenous. Thus, the result cannot be extrapolated to female or older populations. However, this investigation was sufficient to highlight some new methodical aspects of reflection-based PWV measurement methods. There was no invasive monitoring of total or local peripheral resistance or impedance to prove the efficacy of provocation manoeuvres, but the obtained results indicate that physical interventions may alter some reflection-dependent stiffness parameters. As the investigations were performed in one session, the same measurement error may have been hypothesized during the radial central blood pressure monitoring approach during each provocation. Thus, continuous radial monitoring with a transfer factor was chosen rather than an intermittent carotid measurement to avoid placement and angulation-dependent errors. No edge detection software was used for local carotid distensibility calculations, but the assessments were performed by a single, very well trained investigator (Gy.K.) [34]. Although, despite the seemingly large differences between central blood pressure and PWV results derived from different methods, the comparison of absolute values is out of this investigation’s scope, validation studies have formerly been carried out [35,36]. 

The main strength of the investigations is the single-session sequential manner. Accordingly, the repeated distance measurements as the weakest point of PWV calculations were kept off. In addition, both the local carotid stiffness and “two-point” cfPWV were determined to create the possibility for multiple comparisons. Furthermore, an inverse statistical approach and agreement investigation were also carried out to avoid misleading results from primary statistical analysis. 

## 5. Conclusions

In our study, we used several methods to measure different types of arterial stiffness in a young population considered relatively homogeneous regarding age, sex, height and body weight. Our results suggest that PWV detected indirectly with a one-point measurement is not as clinically stable as the classical, two-point, carotid–femoral PWV measurement. Based on our results, the current idea that pulse wave reflection mainly depends on anatomical location is questionable. As the aortic bifurcation of our subjects was considered a fixed anatomical point in our study design, but wave reflection parameters changed significantly with provocation manoeuvres, further studies are needed to assess the clinical relevance of PWV measurements based on reflection. 

In conclusion, it is becoming increasingly urgent to describe a hemodynamic model representative of real-life conditions, allowing the differentiation of various parameters currently referred to collectively as “stiffness”. It seems necessary to separate the parameters indicating the stiffness of large elastic vessels from the complex parameters that depend on peripheral resistance. We believe our findings lay the groundwork for further investigation in this field as a basis for designing more extensive confirmatory studies.

## Figures and Tables

**Table 1:** Comparison of different arterial stiffness parameters at rest, after vasodilation and after occlusion of femoral arteries, expressed as mean and standard deviation.

Parameter	Measurement						*p*-Value		
	Rest		Vasodilation		Occlusion		R vs. V	R vs. O	V vs. O
	Mean	±SD	Mean	±SD	Mean	±SD			
**Oscillometric method**									
Systolic BP (mmHg)	139.20	14.62	133.80	14.21	137.10	16.15	**0.01**	0.38	0.07
Diastolic BP (mmHg)	75.90	10.65	69.40	11.02	75.10	8.12	**0.01**	0.71	**0.04**
Pulse pressure (mmHg)	63.30	10.05	64.40	10.24	62.00	12.46	0.60	0.34	0.23
Augmentation index	5.30	8.74	2.60	4.57	5.80	6.32	0.24	0.80	**<0.01**
Reflection time (msec)	152.90	25.25	160.10	21.81	149.50	19.68	0.09	0.50	**<0.01**
Pulse wave velocity (m/s)	7.46	1.37	7.10	1.18	7.60	1.21	0.05	0.68	**<0.001**
**Ultrasound-based PWV**									
cfPWV (m/s)	5.43	0.79	5.32	0.86	5.28	0.77	0.38	0.27	0.76
**Applanation tonometry**									
Systolic BP (mmHg)	104.30	6.82	102.90	6.02	104.10	6.10	0.20	0.66	**0.04**
Diastolic BP (mmHg)	78.50	5.48	77.90	5.09	77.80	5.05	0.13	0.14	0.48
Pulse pressure (mmHg)	25.90	5.63	25.00	4.58	26.30	5.39	0.11	0.15	**0.01**
HR-corrected Aix	7.30	9.62	−4.70	12.02	4.70	10.05	**<0.01**	0.31	**0.01**
Reflection time (msec)	150.90	20.65	161.10	22.55	149.90	14.99	0.39	0.73	0.13
**Carotid distensibility**									
Distensibility coefficient (10,000/mmHg)	5.07	1.59	5.06	1.99	4.95	1.91	0.88	0.30	0.82

SD—standard deviation, R—rest, V—vasodilation, O—occlusion, BP—blood pressure, cfPWV—carotid–femoral pulse wave velocity, HR—heart rate, Aix—augmentation index, mmHg—millimetres of mercury, msec—millisecond, m/s—metre per second.

## Data Availability

The data presented in this study are available on request from the corresponding author. The data are not publicly available due to privacy concerns.

## Supplementary Materials

The following supporting information can be downloaded at: https://www.mdpi.com/article/10.3390/diagnostics13182897/s1. Table S1: Comparison of different arterial stiffness parameters at rest, after vasodilation, and after occlusion of femoral arteries, expressed as median and interquartile range. Table S2: Intraclass correlation of measured parameters. Table S3: Bland-Altman method comparison of measured parameters. Table S4: Correlation and linear regression analysis of changes in augmentation index, pulse wave velocity and reflection time, during provocation manoeuvres, with oscillometric method.
