# Supplementary material for: One Step Back from Bedside to the Bench—How Do Different Arterial Stiffness Parameters Behave in Relation to Peripheral Resistance?
Source: Diagnostics (Basel). 2023 Sep 9;13(18):2897. doi: 10.3390/diagnostics13182897 (PMC10528548; doi:10.3390/diagnostics13182897)
Supplement: Supplementary file 1 [file diagnostics-13-02897-s001.zip › diagnostics-2402161-supplementary.pdf]

Supplementary Materials

Title

One step back from bedside to the bench – How do different arterial stiffness parameters behave in relation to peripheral resistance?

Authors

Nóra Obajed Al-Ali[1]
Sára Rebeka Tóth[2]
László Váróczy[1]
László Imre Pinczés[1]
Pál Soltész[3]
Zoltán Szekanecz[4]
György Kerekes[5]

Affiliations

[1] Division of Hematology, Department of Internal Medicine, Faculty of Medicine, University of Debrecen, Debrecen, Hungary
[2] Department of Cardiology, Medical Centre, Hungarian Defence Forces, Budapest, Hungary
[3] Division of Angiology, Department of Internal Medicine, Faculty of Medicine, University of Debrecen, Debrecen, Hungary
[4] Department of Rheumatology, Faculty of Medicine, University of Debrecen, Debrecen, Hungary
[5] Division of Intensive Care, Department of Internal Medicine, Faculty of Medicine, University of Debrecen, Debrecen, Hungary

Corresponding Author

Nóra Obajed Al-Ali (obajed.nora@med.unideb.hu)

Keywords

Arterial stiffness; cardiovascular risk; elasticity parameters; distensibility coefficient; pulse wave velocity; oscillometry; applanation tonometry

Parameter	Measurement						p-value		
	Rest		Vasodilation		Occlusion		R vs. V	R vs. O	V vs. O
	Median	IQR	Median	IQR	Median	IQR			
**Oscillometric method**									
Systolic BP (mmHg)	138	9,5	133	11	139	19	**0,01**	0,38	0,07
Diastolic BP (mmHg)	76	11,5	67	14	75	12,5	**0,01**	0,71	**0,04**
Pulse pressure (mmHg)	62	12,5	64	18	58	19	0,60	0,34	0,23
Augmentation index	3,3	7,5	2,5	3,65	7,1	7,4	0,24	0,80	**<0,01**
Reflection time (msec)	147	39,5	155	20	144	21,5	0,09	0,50	**<0,01**
Pulse wave velocity (m/s)	7,3	2,1	7,1	1,45	7,9	1,75	0,05	0,68	**<0,001**
**Ultrasound based PWV**									
cfPWV (m/s)	5,21	0,7	5,22	0,56	5,11	0,76	0,38	0,27	0,76
**Applanation tonometry**									
Systolic BP (mmHg)	105	10,5	105	9	105	8	0,20	0,66	**0,04**
Diastolic BP (mmHg)	81	10	81	10,5	81	9,5	0,13	0,14	0,48
Pulse pressure (mmHg)	25	6,5	25	4,5	26	7,5	0,11	0,15	**0,01**
HR-corrected Aix	4	12,5	-4	16,5	2	11	**<0,01**	0,31	**0,01**
Reflection time (msec)	147	7,75	157,5	12,5	146,5	6,75	0,39	0,73	0,13
**Carotid distensibility**									
Distensibility coefficient (10000/mmHg)	5,3	0,88	5,02	1,44	4,08	2,49	0,88	0,30	0,82

Supplementary Table S1. Comparison of different arterial stiffness parameters at rest, after vasodilation, and after occlusion of femoral arteries, expressed as median and interquartile range
IQR – interquartile range, R – rest, V – vasodilation, O - occlusion, BP – blood pressure, cfPWV – carotid-femoral pulse wave velocity, HR – heart rate, Aix – augmentation index, mmHg – millimetres of mercury, msec – millisecond, m/s – metre per second

Parameter	ICC		
	R vs. V	R vs. O	V vs. O
**Oscillometric method**			
Systolic BP (mmHg)	0,94	0,91	0,95
Diastolic BP (mmHg)	0,81	0,82	0,65
Pulse pressure (mmHg)	0,83	0,94	0,88
Augmentation index	0,35	0,68	0,88
Reflection time (msec)	0,88	0,78	0,94
Pulse wave velocity (m/s)	0,88	0,81	0,95
**Ultrasound based PWV**			
cfPWV (m/s)	0,90	0,88	0,91
**Applanation tonometry**			
Systolic BP (mmHg)	0,97	0,98	0,97
Diastolic BP (mmHg)	0,98	0,97	1,00
Pulse pressure (mmHg)	0,96	0,99	0,97
HR-corrected Aix	0,59	0,68	0,63
Reflection time (msec)	0,19	0,53	0,18
**Carotid distensibility**			
Distensibility coefficient (10000/mmHg)	0,86	0,70	0,81

Supplementary Table S2. Intraclass correlation of measured parameters
ICC – intraclass correlation coefficient for determination of agreement between variables, R – rest, V – vasodilation, O - occlusion, BP – blood pressure, cfPWV – carotid-femoral pulse wave velocity, HR – heart rate, Aix – augmentation index, mmHg – millimetres of mercury, msec – millisecond, m/s – metre per second

Parameter	Bland-Altman Method Comparison								
	R vs. V			R vs. O			V vs. O		
	Mean	-1.96 SD	+1.96 SD	Mean	-1.96 SD	+1.96 SD	Mean	-1.96 SD	+1.96 SD
**Oscillometric method**									
Systolic BP (mmHg)	-5,40	-19,08	8,28	-2,07	-19,40	15,27	3,33	-9,66	16,33
Diastolic BP (mmHg)	-6,47	-23,29	10,36	-0,73	-15,32	13,85	-5,73	-25,07	13,61
Pulse pressure (mmHg)	1,07	-14,03	16,17	-1,33	-11,51	8,84	2,40	-12,26	17,06
Augmentation index	-2,73	-19,90	14,44	0,50	-14,23	15,23	-3,23	-10,28	3,82
Reflection time (msec)	7,20	-23,10	37,50	-3,40	-40,84	34,04	10,60	-9,15	30,35
Pulse wave velocity (m/s)	-0,38	-2,00	1,24	0,11	-1,90	2,13	-0,49	-1,57	0,58
**Ultrasound based PWV**									
cfPWV (m/s)	0,11	-1,08	0,85	-0,15	-1,17	0,86	0,04	-0,87	0,95
**Applanation tonometry**									
Systolic BP (mmHg)	-1,40	-5,44	2,64	-0,20	-3,53	3,13	-1,20	-5,13	2,73
Diastolic BP (mmHg)	-0,53	-3,29	2,23	-0,67	-4,03	2,70	0,13	-1,32	1,59
Pulse pressure (mmHg)	-0,87	-4,71	2,97	0,47	-1,86	2,79	-1,33	-4,86	2,19
HR-corrected Aix	-12,00	-35,10	11,10	-2,60	-21,51	16,31	-9,40	-31,94	13,14
Reflection time (msec)	9,87	-38,76	58,49	7,00	-34,53	48,53	2,87	-54,53	60,27
**Carotid distensibility**									
Distensibility coefficient (10000/mmHg)	-0,01	-2,31	2,28	-0,12	-3,15	2,90	0,11	-2,77	2,99

Supplementary Table S3. Bland-Altman method comparison of measured parameters
R – rest, V – vasodilation, O - occlusion, SD - standard deviation, BP – blood pressure, cfPWV – carotid-femoral pulse wave velocity, HR – heart rate, Aix – augmentation index, mmHg – millimetres of mercury, msec – millisecond, m/s – metre per second

Parameter	Correlation		Linear regression			
	**Spearman r**	**p**	**R^2**	**F**	**Y=**	**p**
PWV^v-PWV^r vs. Aix^v-Aix^r	-0,1728	0,5339	0,05702	0,7861	-2,528*X - 3,694	0,3914
PWV^o-PWV^v vs. Aix^o-Aix^v	0,2421	0,3806	0,08333	1,182	1,898*X + 2,297	0,2967
PWV^o-PWV^r vs. Aix^o-Aix^r	0,3358	0,2194	0,001251	0,01628	-0,2581*X + 0,5293	0,9004
PWV^v-PWV^r vs. RT^v-RT^r	-0,9507	<0,0001	0,9231	156,0	-17,95*X + 0,3779	<0,0001
PWV^o-PWV^v vs. RT^o-RT^v	-0,9059	<0,0001	0,8171	58,09	-16,65*X - 2,385	<0,0001
PWV^o-PWV^r vs. RT^o-RT^r	-0,9629	<0,0001	0,9664	373,7	-18,24*X - 1,333	<0,0001

Supplementary Table S4. Correlation and linear regression analysis of changes in augmentation index, pulse wave velocity and reflection time, during provocation manoeuvres, with oscillometric method

PWV – pulse wave velocity, Aix – augmentation index, RT – reflection time, ^{R} – rest, ^{V} – vasodilation, ^{O} – occlusion, r – correlation coefficient, R^2 – coefficient of determination, F – explained variance, Y= – equation of the linear regression line
